# Application of Nanomaterials in Pulmonary Drug Delivery

**DOI:** 10.3390/pharmaceutics18091057

**Published:** 2026-08-26

**Authors:** Ravi Maharjan, Ivana D’Angelo

**Affiliations:** 1 Laboratory of Biopharmaceutics, Yonsei University, Songdo, Incheon 21983, Republic of Korea; 2 Department of Environmental, Biological and Pharmaceutical Sciences and Technologies, University of Campania “Luigi Vanvitelli”, 81100 Caserta, Italy

## 1. Introduction

The respiratory tract is one of the most fascinating yet challenging routes for drug delivery. Pulmonary delivery has developed from its historical use in treating localised asthma and chronic obstructive pulmonary disease (COPD) into a sophisticated technique for delivering systemic therapy and complex biopharmaceuticals, including peptides, proteins, and nucleic acids. The lung’s unique anatomical features, particularly its large surface area (~70–100 m^2^) and thin epithelial barrier of the alveolar region, create an ideal environment for rapid drug absorption and high bioavailability. Due to the avoidance of first-pass hepatic metabolism and relatively low enzymatic activity compared to the gastrointestinal tract, the lungs are a preferred target for labile bioactives [1,2]. Effective pulmonary therapy is hindered by a series of substantial physiological and anatomical challenges. The body has developed highly efficient mechanisms within the respiratory system to clear inhaled foreign particles. Factors such as mucociliary escalator in the conductive airways, phagocytic activity of alveolar macrophages in the lung, complex extracellular matrix, and presence of lung surfactant can significantly alter the fate of inhaled drug delivery systems. Researchers in academia and the pharmaceutical industry are now focusing on nanotechnology to overcome these challenges. The physicochemical properties of nanomaterials, such as size, shape, surface charge, and aerodynamic diameter, can be adjusted to improve lung deposition and facilitate passage across cellular and extracellular barriers.

Research outside of this Special Issue continues to encounter challenges with regard to the long-term safety of nanomaterials, including potential lung accumulation, immunogenicity, and chronic toxicity. Studies have shown a growing trend towards personalised inhalation therapy, where the connection between a patient’s respiratory physiology, delivery device, and nanoparticle formulation is carefully adjusted to ensure precise dosing [3]. The advent of smart material is redefining boundaries within respiratory medicine, able to respond to internal stimuli, including pH shifts in infected lung tissue and enzymatic triggers [4]. This Special Issue of Pharmaceutics, titled “Application of Nanomaterials in Pulmonary Drug Delivery”, was conceived to highlight these developments. This collection of 10 published papers serves as a testament to the multidisciplinary efforts currently shaping the field, encompassing innovative synthesis methods and advanced in vitro models for evaluating therapeutic efficacy.

## 2. Overview of Published Work

The research and review articles in this Special Issue offer a comprehensive overview of how nanomaterials are being used to overcome particular challenges in pulmonary therapy. Contributions presented here showcase different technological methods employed by authors to enhance the compatibility and efficacy of nanoparticles within the lung environment.

### 2.1. Overcoming the Mucus Barrier in Gene and Peptide Delivery

The treatment of diseases like cystic fibrosis or chronic bronchitis is complicated by a thick, viscous mucus layer that traps and prevents therapeutic agents from being cleared. Arca et al.’s contribution addresses this concern by designing carbon dots (CDs) with mucoinert properties. The authors functionalised CDs with maleamic acid groups using a solvothermal treatment of citric acid and branched polyethyleneimine to create cationic nanoparticles with a tunable charge. A 1000- to 10,000-fold increase in transfection efficiency was observed in mucus-producing Calu-3 cell models grown at the air–liquid interface. Functionalised CDs provide a promising platform for pulmonary gene therapy by enabling the penetration of pDNA through a thick mucus layer.

A contribution by Cresti et al. examines the encapsulation of antimicrobial peptide SET-M33 within polymeric nanoparticles as part of a parallel initiative to protect sensitive bioactives. A biocompatible system was developed, using PLGA conjugated with PEG, which protects the peptide from airway barriers and enables sustained release. The system displayed prolonged antibacterial activity against Pseudomonas aeruginosa in both planktonic and biofilm forms, with the additional benefit of substantially lower cytotoxicity compared to the free peptide. In vivo studies further confirmed the system’s capacity to reduce toxic effects of the peptide, representing a crucial step towards clinical application of peptide-based inhaled treatments.

### 2.2. Targeted Delivery and Organ Tropism

Targeting specific lung regions with nanoparticles or using the lung as a reservoir for systemic drug delivery is a holy grail of nanomedicine. A contribution by Ye et al. provides profound insights into how the external chemistry of mesoporous silica nanoparticles (MS NPs) affects their organ tropism. The study discovered that MS NPs usually accumulate in the liver, but those enriched with –SH groups exhibit a distinct preference for the lungs. The shift is attributed to the high affinity of these lung-preferred nanocarriers for albumin, which triggers targeting and trafficking of tumour cells. Using thioether-bridged deformable hollow mesoporous organosilica nanoparticles (HSMONs) loaded with gambogic acid, the particles can have a strong effect against lung cancer that has spread, highlighting the potential of surface chemistry to overcome distribution problems.

### 2.3. Sustainable Synthesis and Anti-MDR Strategies

A sustainable green synthesis method is introduced by Alabbosh et al. in their contribution to address the global health crisis of multidrug-resistant (MDR) infections. The authors biosynthesised pH-responsive chitosan–silver hybrid nanoparticles (CS–Ag HNPs) using Pseudomonas fluorescens bacterial extracts. Nanoparticles loaded with ciprofloxacin exhibited excellent aerodynamic characteristics, including a mass median aerodynamic diameter (MMAD) of 2.6 μm, which is well-suited for deep lung deposition. A hybrid system led to a 4-fold rise in antimicrobial activity against MDR Pseudomonas aeruginosa and Klebsiella pneumoniae, accompanied by significant biofilm disruption. The work showcases the potential of integrating natural materials with cutting-edge fabrication methods to produce environmentally friendly, high-performance pulmonary treatments.

### 2.4. Controlled Release and Formulation Optimisation

Chronic inflammation in conditions like asthma and COPD requires long-acting formulations to reduce dosing frequency and improve patient compliance. A contribution by Craparo et al. details the production of inhalable beclomethasone dipropionate (BDP) dry powders using novel PHEA-based copolymers. The authors optimised the spray–drying process to produce microparticles, resulting in a controlled release profile of BDP that was 3× greater than that of commercial formulations like Clenil. Polymer-based nanocarriers were highly efficient in counteracting inflammatory markers such as IL-6 and IL-8 in bronchial and alveolar epithelial cells, thereby significantly enhancing the therapeutic index of established corticosteroids. A comprehensive overview of lipid and polymer-based nanocarriers is provided by Costabile et al. The review offers a critical evaluation of the intricate connection between the physiochemical characteristics of the payload, inhalation device, and biological obstacles of the lung, thus outlining a framework for the future advancement of effective nanoparticulate carriers for inhalation.

## 3. Future Perspectives

The findings of this Special Issue suggest that we are entering an era of intelligent pulmonary delivery. Key themes are likely to shape the research landscape in the years to come. The integration of biomimetic strategies, including the use of nanoparticles coated with cell membranes such as those from macrophages, neutrophils, or cancer cells, holds immense potential for treating lung cancer and severe infections. Implementing Quality by Design principles from the outset is essential for reproducible and scalable production of complex nano-formulations. A systematic understanding of how process parameters such as stirring speed or stabiliser concentration affect nanoparticle size and loading is crucial for achieving clinical success [5].

We expect future studies focused on the systems that are multi-targeted and responsive to stimuli. Releasing a payload only in the presence of specific bacterial enzymes or within the acidic microenvironment of a tumour reduces systemic side effects and increases local efficacy. The development of complex in vitro models, including organ-on-a-chip and advanced air–liquid interface culture, continues to reduce our dependence on animal models, providing accurate data on human lung responses [6]. The editors wish to express their sincere gratitude to the authors for their excellent submissions and to the reviewers for their thorough assessment of these papers. The Special Issue’s success reflects the vibrant and innovative community dedicated to advancing respiratory medicine. The collection offers a valuable resource and promotes further progress in the application of nanomaterials for pulmonary drug delivery.

## References

[1] He S., Gui J., Xiong K., Chen M., Gao H., Fu Y. (2022). A roadmap to pulmonary delivery strategies for the treatment of infectious lung diseases. J. Nanobiotechnol..

[2] Liu L., McClements D.J., Liu X., Liu F. (2024). Overcoming biopotency barriers: Advanced oral delivery strategies for enhancing the efficacy of bioactive food ingredients. Adv. Sci..

[3] Zhou S., Ran S., Zhang F., Ma H., Jiang C., Li S., Zhang Q. (2026). Advanced Multiscale Inhalation Platforms for Treatment of Pulmonary Diseases. Int. J. Nanomed..

[4] Liang D., Wang H., Jiang Y., Zhang Z., Zhou T., Ge S., Tan S., Qin K., Wang Y., Lin X. (2026). Smart biomaterials for skeletal aging repair and regeneration. Bone Res..

[5] Gonçalves R.S. (2026). Artificial Intelligence in Nanopharmaceutical Development: From Predictive Design to Clinical Translation. Pharmaceutics.

[6] Yang S., Zhang T., Ge Y., Niu Y., Chen M., Yin L., Pu Y., Chen Z., Gu Z., Liang G. (2026). Organ-on-a-chip toxicology. Innovation.

